# Evaluation of an Imine-Linked Polymer Organic Framework for Storage and Release of H_2_S and NO

**DOI:** 10.3390/ma16041655

**Published:** 2023-02-16

**Authors:** Sílvia Carvalho, João Pires, Cristina Moiteiro, Moisés L. Pinto

**Affiliations:** 1 CERENA, Departamento de Engenharia Química, Instituto Superior Técnico, Universidade de Lisboa, Campus Alameda, 1049-001 Lisboa, Portugal; 2 CQE, Centro de Química Estrutural, Institute of Molecular Sciences, Departamento de Química e Bioquímica, Faculdade de Ciências, Universidade de Lisboa, Campo Grande, 1749-016 Lisboa, Portugal

**Keywords:** porous organic cages, gas adsorption, delivery vehicles, NO/H_2_S release

## Abstract

Hydrogen sulfide (H_2_S) and nitric oxide (NO) are especially known as toxic and polluting gases, yet they are also endogenously produced and play key roles in numerous biological processes. These two opposing aspects of the gases highlight the need for new types of materials to be developed in addition to the most common materials such as activated carbons and zeolites. Herein, a new imine-linked polymer organic framework was obtained using the inexpensive and easy-to-access reagents isophthalaldehyde and 2,4,6-triaminopyrimidine in good yield (64%) through the simple and catalyst-free Schiff-base reaction. The polymeric material has microporosity, an *A*_BET_ surface area of 51 m^2^/g, and temperature stability up to 300 °C. The obtained 2,4,6-triaminopyrimidine imine-linked polymer organic material has a higher capacity to adsorb NO (1.6 mmol/g) than H_2_S (0.97 mmol/g). Release studies in aqueous solution showed that H_2_S has a faster release (3 h) from the material than NO, for which a steady release was observed for at least 5 h. This result is the first evaluation of the possibility of an imine-linked polymer organic framework being used in the therapeutic release of NO or H_2_S.

## 1. Introduction

The intensive research on porous materials has led to a high diversity of materials, with numerous applications ranging from gas storage and separation to water purification and biomedical applications. However, the role of porous materials dates to ancient Egyptian times, when they used porous charcoal for indigestion. Yet, it was only in the 18–19th centuries that the adsorption properties of porous materials began to be studied, and investigation of other materials such as zeolites and mesoporous silica started to emerge [1]. Currently, the design of materials with high complexity, such as porous organic polymers (POPs), metal–organic frameworks (MOFs), porous coordination cages (PCCs), porous organic cages (POCs), and hydrogen-bonded organic frameworks (HOFs) shows that much work is still needed in porous materials.

Purely organic materials do not present some weaknesses of other hybrid materials, such as MOFs, in which the presence of a metal center and relatively labile coordination bonds may cause stability and toxicity problems when exposed to some conditions [2]. These issues are even more significant when reactive gases such as nitric oxide (NO) and hydrogen sulfide (H_2_S), which coordinate to open metal sites of MOFs, are investigated [2]. Inorganic materials such as zeolites and titanosilicates have been chosen for adsorption studies involving NO [3,4,5,6] and H_2_S [7], especially for biomedical applications, due to their biocompatibility and stability. The use of POPs is a possible alternative due to their high chemical and physical stability, tailorable backbone allowing the introduction of specific organic functionalities, and solubility facilitating solution-based processing [8]. They are versatile materials with applications in diverse areas, such as biomedical [9], sensing [10], gas storage and separation [11,12,13,14,15,16], and catalysis [17].

Several synthetic methodologies have been used in the POP synthesis, with the Schiff-base reaction standing out. Because of its versatility, this reaction is widely used. It involves the reaction of amines (e.g., 1,3,5-triazine-2,4,6-triamine (melamine), 1,4-bis-(2,4-diamino-1,3,5-triazine)-benzene,1,2,4,5-benzenetetramine, 2,3,6,7,10,11-hexaaminotriphenylene,2,3,6,7,14,15-hexaaminotriptycene, and 2,4,6-triaminopyrimidine) with aldehyde or ketone compounds (e.g., terephthalaldehyde, isophthalaldehyde, thieno[2,3-b]thiophene-2,5-dicarboxaldehyde, 1,3,5-benzenetricarboxaldehyde, and 2,4,6-trihydroxybenzene-1,3,5-tricarbaldehyde) without expensive catalysts [8,16,18,19,20,21,22,23,24,25,26,27,28]. The obtained porous network materials may be crystalline (crystalline covalent organic frameworks (COFs)) or amorphous (imine-linked polymer organic frameworks (i-POFs)). Obtaining one or the other depending on the control of the thermodynamic equilibrium [29].

**Figure 1:**
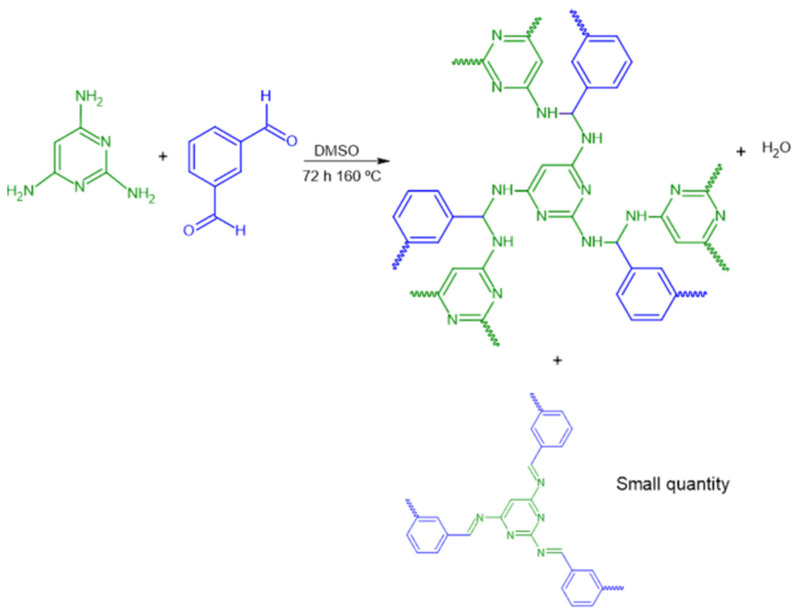
Schematic representation of the synthetic path.

In this work, we synthesized and characterized a 2,4,6-triaminopyrimidine-based i-POF using the condensation of the 2,4,6-triaminopyrimidine with the divalent isophthalaldehyde monomers, forming an intermediate imine or Schiff base (Figure 1). The fact that 2,4,6-triaminopyrimidine has not been much explored as a building block in i-POFs synthesis, allied with its accessible price, inspired us to use it in this work. The H_2_S/NO adsorption capacities were evaluated in order to assess the storage potential of the synthesized material. Additionally, their H_2_S/NO release in aqueous solution at physiological pH was performed as a first approach to assess the potential for therapeutic applications as NO/H_2_S carriers in the future.

## 2. Materials and Methods

### 2.1. General Remarks

Isophthalaldehyde (97%, Sigma-Aldrich, Spain), 2,4,6-triaminopyrimidine (97%, Sigma-Aldrich, Spain), sodium sulfide nonahydrate (≥99.99%, Sigma-Aldrich, Spain ), sodium dithionite (≥82%, RT, Sigma-Aldrich, Spain ), DTNB [5,5′-dithiobis(2-nitrobenzoic acid), >98%, TCI, Belgium], Teflon particle size 35 μm (Sigma-Aldrich, Spain), and hemoglobin human lyophilized powder (Sigma-Aldrich, Spain) as reagents, as well as dimethyl sulfoxide (DMSO, Honeywell Reagents, Germany), acetone (Honeywell Reagents, Germany), tetrahydrofuran (THF, Honeywell Reagents, Germany), and dichloromethane (Honeywell Reagents, Germany) as solvents, were used without further purification. Nitric oxide gas (99.99%) and hydrogen sulfide (99.5%) gases were purchased from Air Liquide, Portugal.

### 2.2. i-POF Synthesis

The i-POF was synthesized following a reported procedure [20], albeit using different amine and aldehyde. Briefly, 2,4,6-triaminopyrimidine (311 mg, 2.5 mmol), isophthalaldehyde (500 mg, 3.7 mmol), and DMSO (15.5 mL) were added into a Schlenk flask. The mixture was degassed with N_2_ before being heated at 160 °C for 72 h in an inert atmosphere. After cooling to room temperature, the precipitate was filtered and washed with acetone, THF, and dichloromethane. The obtained solid was dried using a vacuum line to afford a yellow powder in 64% yield. Elemental analysis: calculated for C_46_H_56_N_14_.2H_2_O, C, 66.0%; H, 6.74%; N, 23.4%; found, C, 57.8%; H, 4.3%; N, 23.5%. The elemental analysis of i-POF does not strictly match the structure proposed (Figure 1) because the imine produced in the first step subsequently suffers attack by another primary amine, resulting in an organic polymer comprising aminal with residual imine groups [30].

### 2.3. i-POF Characterization

The Fourier-transform infrared (FTIR) spectra of the i-POF and isophthalaldehyde were recorded in KBr pellets between 4000 and 400 cm^−1^ (64 scans; 4 cm^−1^ resolution) using a Nicolet 6700 FTIR spectrometer. The scanning electron microscope (SEM) images for the analysis of the powder morphology were acquired using a Phenom ProX G6 desktop SEM-Thermoscientific using 55 kV as accelerating voltage. The samples were sputtered-coated with a gold/palladium alloy (80/20 wt.%) (5–10 nm 110 thick). The material’s XRD pattern was obtained with a powder X-ray diffractometer (Pan Analytical PW3050/60X’Pert PRO) in the 5°–60° (2θ) range with CuKα radiation (λ = 0.15406). Elemental analysis was carried out in a CHNS Analyzer (Thermofinnigan Flash, EA, 1112 series, USA). A Setaram (mod. TG-DSC 111) equipment coupled with differential scanning calorimetry (TG-DSC) was used in the thermal analysis studies. Experiments were carried out under air flux with a temperature ramp of 5 °C/min from ambient to 600 °C. The nitrogen gas adsorption–desorption isotherm was obtained at low temperature (−196 °C) in a constant-volume adsorption automated apparatus (Quantacrome, Nova 2200e, Boynton Beach, FL, USA), using about 50 mg of sample. The sample was degassed under a vacuum of 0.133 Pa at 120 °C for 2.5 h. The N_2_ isotherm data were used to estimate the apparent area, *A*_BET_, and then to evaluate it through the BET equation (0.05 < *p/p*^0^ < 0.15) and ISO 92777 [31,32]. The microporosity was analyzed with the NLDFT (nonlocal density functional theory) model, using the N_2_ silica equilibrium transition kernel at 77 K based on a cylindrical pore model provided by NovaWin version 10.0 software.

### 2.4. H_2_S and NO Adsorption Studies

A volumetric apparatus and a gravimetric apparatus were used for the determination of the H_2_S and NO gas–solid adsorption data, respectively. The sample temperature (25 °C) was maintained with a water bath (Sub Aqua 2 Plus, Grant). A schematic representation of the apparatus is shown in [33]. The determination of the H_2_S isotherm was not possible due to slow kinetics. Therefore, only the maximum adsorption is shown, as described in [34]. The NO adsorption was measured for up to 20 h in order to make a more accurate comparison with the H_2_S adsorption. Before measurement, the sample (~60 mg) was subjected to a vacuum better than 10^−2^ Pa, for 2.5 h at 120 °C for outgassing.

### 2.5. H_2_S and NO Release Studies in Aqueous Solution

For the aqueous solution H_2_S/NO kinetic release studies, the i-POF was mixed with Teflon particles in a wt.% ratio of 75:25 (sample: Teflon) and converted into disc pellets with 5 mm diameter in order to avoid sample dispersion in the liquid. The prior outgassing was conducted using the same conditions as the adsorption studies. The loading of the material with H_2_S/NO used the procedure already described in [34,35].

The DTNB and oxyhemoglobin assays were used for the H_2_S and NO release studies, respectively. These two methodologies are appropriate for these studies since they allow detecting the presence of H_2_S and NO at physiological pH (pH = 7.2). The preparation of the solutions and the detailed experimental procedures are described elsewhere [34,35]. Briefly, the DTNB methodology is based on the absorbance (408 nm) of the anion 5-thio-2-nitrobenzoate formed in the reaction between DTNB and H_2_S [36]. The calibration curve and corresponding spectra of the DTNB method are presented in the (Supplementary Materials Figure S1). The oxyhemoglobin assay is based on the oxidation of NO to nitrate by oxyhemoglobin (HbO_2_). The reaction begins with methemoglobin (metHb), which absorbs at 406 nm. A shift to 415 nm (absorbance of HbO_2_) is observed, allowing the NO to be quantified [37].

The experiments were conducted by adding the H_2_S/NO loaded pellets to the DTNB or hemoglobin solutions, respectively. The first spectrum was acquired after 2 min of the pellet addition, followed by 15 to 30 min intervals until no changes were observed in the spectra, or all the hemoglobin was consumed. The absorbance spectra were recorded using a UV/Vis spectrophotometer (Genesys 10S UV/Vis spectrophotometer from Thermo Scientific Blank, Waltham, MA, USA) at room temperature. The absorbances were measured in the 250–500 nm range for the DTNB method and 350–700 nm for the oxyhemoglobin assay. The experimental conditions used in this study are shown in Table 1.

## 3. Results and Discussion

### 3.1. i-POF Characterization

**Figure 2:**
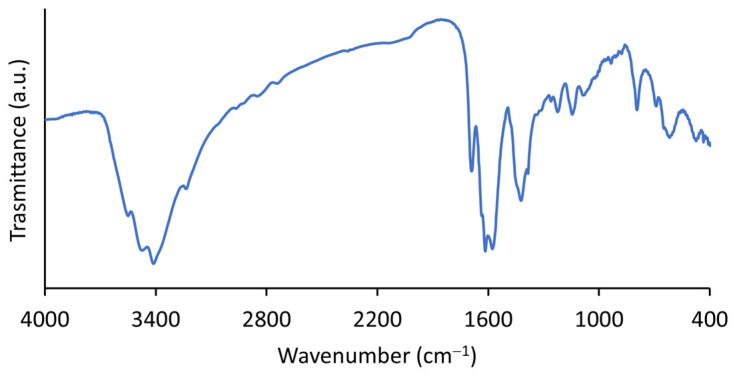
FTIR spectra of the i-POF.

In the FTIR spectra of i-POF (Figure 2), the band at 1700 cm^−1^, corresponding to the C=O stretching of the aldehyde group, had a much lower intensity than that observed in the isophthalaldehyde spectra (Figure S2), suggesting that most of the aldehyde group reacted and that the residual band was from terminal aldehyde functional groups. To reinforce this statement, the aldehyde C–H stretching and bending bands at 2870 cm^−1^ and 1380 cm^−1^, respectively, nearly disappeared in the i-POF spectra [38]. It is also possible to observe N–H stretching at 3406 and 3230 cm^−1^ (free and hydrogen-bonded, respectively) and N–H bending at 1620 cm^−1^. The bands at 1577 cm^−1^ and 1420 cm^−1^ correspond to pyrimidine ring C=N stretching and residual imine and C–N stretching, respectively [8,23].

**Figure 3:**
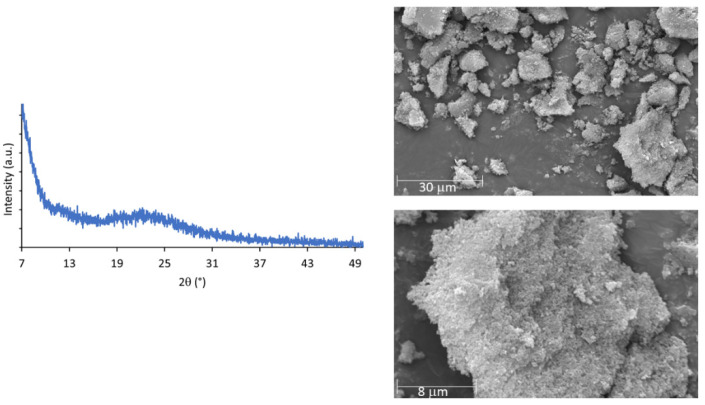
Powder XRD patterns (**left**) and SEM images (**right**) of the i-POF.

The TG-DSC curve (Figure S3) and powder XRD patterns of i-POF are similar to other i-POF materials, particularly the related 2,4,6-triaminopyrimidine- and melamine- i-POFs, with the material being stable up to 300 °C [23] and its amorphous characteristics (Figure 3 left) confirmed by the broad band at 22° observed in the powder XRD patterns. The corresponding d spacing value was 0.4 nm [8,22,23,30]. Scanning electron microscopy (SEM) (Figure 3—right) revealed aggregated particles of variable size with no defined morphology [20,23].

**Figure 4:**
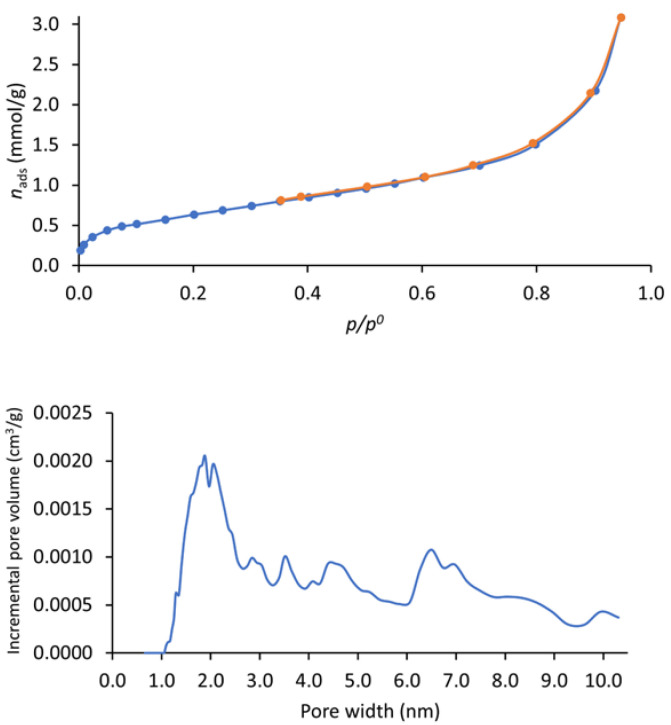
Nitrogen adsorption (in blue)–desorption (in orange) (**top**)and corresponding pore size distribution curves (**down**) of i-POF.

The N_2_ adsorption–desorption isotherm at −196 °C (Figure 4—top) shows a typical type I + II profile [20,23]. The *A*_BET_ surface area was 51 m²/g, a value much lower than observed for similar polymers, which have typical values of ~200–1000 m²/g [20,21,22,24,33]. Analyzing these results, it is possible to understand aspects influencing the *A*_BET_ of these types of materials. The first is the reaction temperature; for instance, in the melamine-based polymer, the *A*_BET_ surface area decreased from 1133 m²/g [20] to 86.15 m²/g [22] due to the unordered connections between the building units caused by the lowering of the temperature. This was also observed for the related 2,4,6-triaminopyrimidine-based i-POF, for which a temperature of 150 °C was used, leading to an *A*_BET_ value of 203 m²/g. The second is the modification of the positional isomer in the monomer from *para* to *meta* position. This factor led to a reduction in the *A*_BET_ due to the obtention of a more distorted structure. Accordingly, we decided to conjugate those two factors with the purpose of obtaining an i-POF with lower *A*_BET_, thus limiting the H_2_S/NO adsorption capacity. In doing so, it is possible to use a higher quantity of material without running the risk of toxicity caused by the release of excess gas in therapeutic applications. The pore distribution analysis (Figure 4—down) shows that most pores in our sample had widths of ~2 nm.

### 3.2. H_2_S and NO Adsorption by i-POF

i-POFs have been investigated in different areas with promising results. Recently, a triazine-based porous organic polymer was used for the capture of volatile iodine [24]. Furthermore, a 4,4′-biphenyldicarboxaldehyde m-phenylenediamine Schiff-base magnetic polymer revealed efficiency in the removal of phenanthrene and 9-phenanthrol [25]. The related melamine and 2,4,6-triaminopyrimidine-based i-POFs were investigated as catalysts [23,39], as well as for the removal of copper [30] and organic pollutants [22]. Concerning gas adsorption, few studies exist, thus highlighting the necessity to carry out other studies [14,21]. In fact, a study concerning the adsorption of H_2_S by organic polymer used a microporous triazine-based ionic hyper-crosslinked polymer not obtained by the Schiff-base reaction [40].

As already discussed, most of the pores of i-POFs have widths of ~2 nm, a value that is higher than the kinetic diameters of H_2_S (0.36 nm) and NO (0.317 nm) [18,41]; thus, the molecules may access the pores. Nevertheless, the adsorption of both gases was slow. Due to experimental limitations, the isotherm of the H_2_S could not be determined, yet it was possible to determine the maximum adsorption capacity, which had a value of 0.97 mmol/g. This value is of the same order of magnitude as the value obtained for the best PCL/4A zeolite composite [34] and higher than that observed for the mesoporous SBA-15, which had a higher *A*_BET_ and higher pore opening [7]. The presence of amines in the i-POF structure may contribute to the adsorption process since the amine affinity for H_2_S is well known. Yet, it is lower than for microporous triazine-based ionic hyper-crosslinked polymers, which showed values of 3.91–4.85 mmol/g. This difference may be attributed not only to structural factors and a higher *A*_BET_, but also to the presence of charges, which may increase the interaction with H_2_S [40].

**Figure 5:**
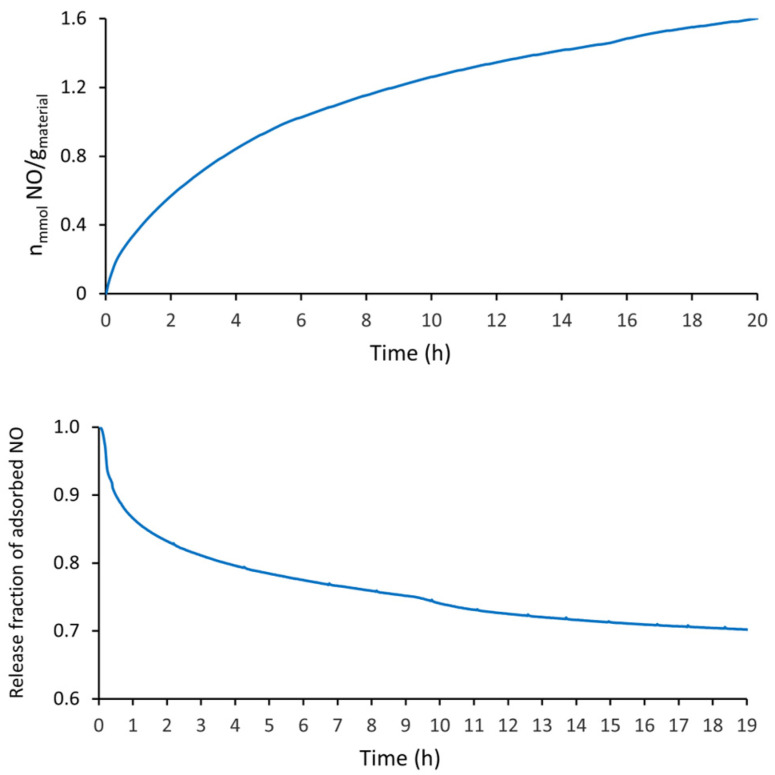
NO adsorption capacity of i-POF. Kinetic profiles at 80 kPa (**top**); NO release profiles under vacuum (**bottom**).

The quantity of NO adsorbed by the i-POF (at the same experiment time—20 h) was about 1.6 times higher than that of H_2_S, as can be seen in Figure 5. This may be related to its smaller kinetic diameters and its high reactivity, which may have increased the chemical adsorption. This fact may be corroborated by the kinetic desorption curves (Figure 5—bottom), which showed that only a small percentage of NO (~30%) was desorbed from the structure after 20 h of vacuum.

The amount of NO adsorbed by the i-POF was greater than that of the PCL/4A composites and comparable with the 4A zeolite (at 20 h). On the other hand, the amount of NO release was intermediate between the PCL/4A composite (15%) and 4A zeolite (38%) [34]. Concerning the NO adsorption by MIP-177 and vitamin B_3_ MOF, the i-POF had a lower adsorption and NO release capacity [42,43].

The i-POF was loaded with H_2_S and NO, and then left under an inert atmosphere for 3 weeks; after that, the material was added to a DTNB and hemoglobin solution. It was possible to observe a change in the color of the solution, indicative of H_2_S and NO being released from the material. This is an important result, especially for therapeutic applications, since it demonstrates the stability of these molecules inside the pore.

### 3.3. H_2_S and NO Release by i-POF in the Liquid Phase

**Figure 6:**
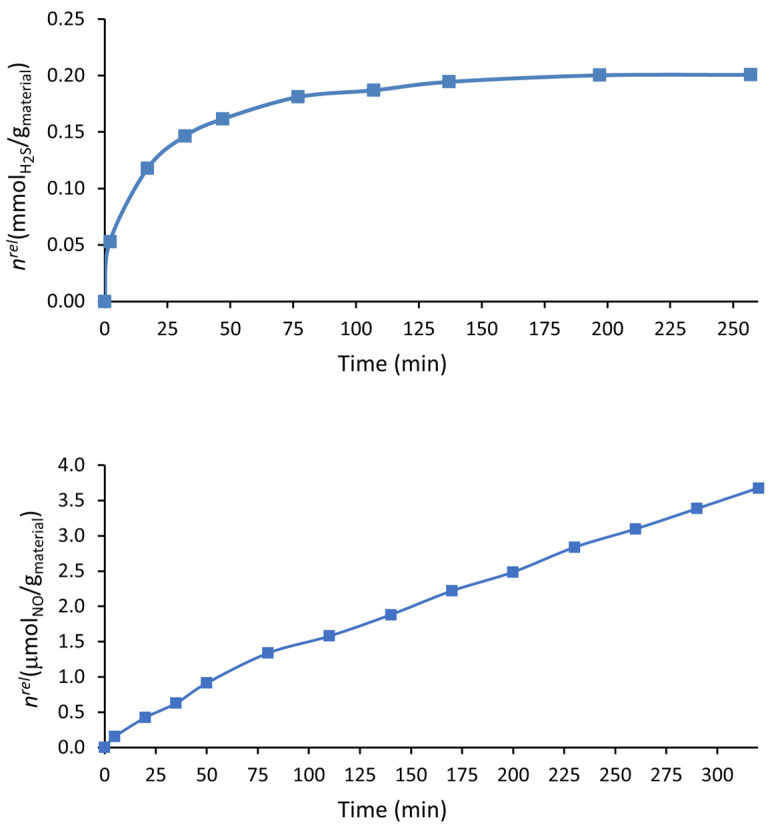
H_2_S release profile of the H_2_S-loaded i-POF material in aqueous solution using the DTNB method (**top**). NO release profiles in liquid phase from NO-loaded i-POF material using the oxyhemoglobin assay (**bottom**).

An essential condition for porous materials to be used as gasotransmitter vehicles is their release profile at physiological pH. The ideal release profile depends on the application; for therapeutic applications, a slow release rate is usually needed [15]. As seen in Figure 6, the H_2_S and NO release profiles of i-POF in aqueous solution were different. While the H_2_S had a rapid release in the first ~50 min, releasing 80.5% of the gas, followed by a continuous release of a small quantity of gas until ~200 min (~3 h), NO had a continuous steady release for at least 320 min (~5 h). Other differences lie in the quantity of gas released by the material, which was more than 50 times higher for H_2_S, and in the percentage of the gas released (against the total adsorbed), which, although low for both gases (Table 2), was almost negligible for NO. This fact may be explained by the degradation of the gases into the i-POF, which is expected to occur at a higher rate for NO due to its higher instability. It is well known that NO is rapidly oxidized to NO_2_^−^, followed by its dimerization to N_2_O_4_. It should be noted, however, that this value for NO would be higher as the release of gas continues. Furthermore, the solubility of the gases in water may influence these results; in fact, the solubility of NO at 25 °C in an aqueous solution of 1.94 × 10^−6^ mol·cm^−3^ is much lower than that of H_2_S (2.00 × 10^−3^ mol·cm^−3^) [44,45].

Comparing these results with other results obtained using the oxyhemoglobin assay on other porous materials in the literature, they are similar to or better than the 4A/PCL composite (the porous material with the best results). The i-POF showed a similar NO release profile, yet a longer release was obtained for the H_2_S [34]. i-POF also had a similar NO release profile to the vitamin B_3_ MOF [42].

## 4. Conclusions

A novel nitrogen-rich 2,4,6-triaminopyrimidine-based i-POF was synthesized from organic functional precursors, 2,4,6-triaminopyrimidine and isophthalaldehyde, through the Schiff-base reaction. From an industrial viewpoint, this catalyst-free condensation via a one-step reaction from inexpensive and commercially available monomers, 2,4,6-triaminopyrimidine and meta-dialdehyde, was carried out on laboratory scale without any difficulty. The synthesis procedure is highly efficient and easy to scale up. The precise synthetic control was achieved and resulted in 2,4,6-triaminopyrimidine-based i-POF microporous structure. The reaction was under kinetic control, resulting in the formation of disordered and, as a result, amorphous materials. All the characterization results (FTIR, SEM, and DRX) were very similar for the melamine-based and related 2,4,6-triaminopyrimidine-based polymers.

The H_2_S adsorption capacity of i-POF was better than some common materials such as SBA-15, while the NO adsorption capacity was similar to that of 4A zeolite. Compared with the PCL/4A composite, the NO adsorption capacity of i-POF was higher, while the H_2_S adsorption capacity showed a similar behavior.

Regarding the H_2_S and NO aqueous releases by the i-POF, the NO showed a similar release profile, while the H_2_S had a longer release time than the PCL/4A composite. Although it had a lower NO adsorption capacity than the MOF, it had a similar release profile.

This study investigated the H_2_S /NO adsorption and release capacities of an i-POF material. It showed properties are competitive with other promising materials; hence, it may be exploited as a H_2_S/NO vehicle. These results can inspire the design of i-POF materials for the adsorption and release of H_2_S and NO in real therapeutic applications.

## Figures and Tables

**Table 1:** Experimental conditions used in the H_2_S/NO release studies.

Method	m_pellets_ (mg)	Volume (mL)
DTNB	6.7	50
Oxyhemoglobin	3.4	3

**Table 2:** H_2_S and NO released by i-POF in aqueous solution at pH 7.2 at room temperature determined using the DTNB method and oxyhemoglobin assay.

Gas	gas_released_ (μmol_gas_/g_material_)	gas_released_ (%)	*t*_max_ (min)
H_2_S	200.7	20	200
NO *	3.7	0.2	320

* The data correspond to the time of the experiment at which the release continued.

## Supplementary Materials

The following supporting information can be downloaded at https://www.mdpi.com/article/10.3390/ma16041655/s1: Figure S1. UV/Vis spectra of the DTNB (decreasing) and 5-thio-2-nitrobenzoate (increasing) (left) and calibration curve (right) obtained using a Na_2_S solution; Figure S2. FTIR spectrum of isophthalaldehyde; Figure S3. Thermogravimetry (blue line) and differential scanning calorimetry (orange line) of the studied material.
