# Supplementary material for: Evaluation of an Imine-Linked Polymer Organic Framework for Storage and Release of H2S and NO
Source: Materials (Basel). 2023 Feb 16;16(4):1655. doi: 10.3390/ma16041655 (PMC9967787; doi:10.3390/ma16041655)
Supplement: Supplementary file 1 [file materials-16-01655-s001.zip › materials-2170381-supplementary.pdf]

Supplementary Materials

Evaluation of an imine-linked polymer organic framework for storage and release of H_2S and NO

Sílvia Carvalho[1,2*], Cristina Moiteiro[2], João Pires[2], and Moisés L. Pinto[1*]

[1]CERENA, Departamento de Engenharia Química, Instituto Superior Técnico, Universidade de Lisboa, Campus Alameda, 1049-001 Lisboa, Portugal

[2]CQE, Centro de Química Estrutural, Institute of Molecular Sciences, Departamento de Química e Bioquímica, Faculdade de Ciências, Universidade de Lisboa, Campo Grande, 1749-016 Lisboa, Portugal.

[*] Correspondence: silvia.carvalho@tecnico.ulisboa.pt (S.C.); moises.pinto@tecnico.ulisboa.pt (M.L.P)

S1. H_2S release in aqueous solution

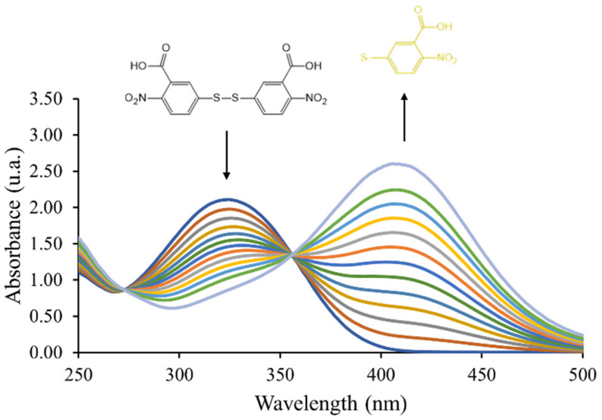

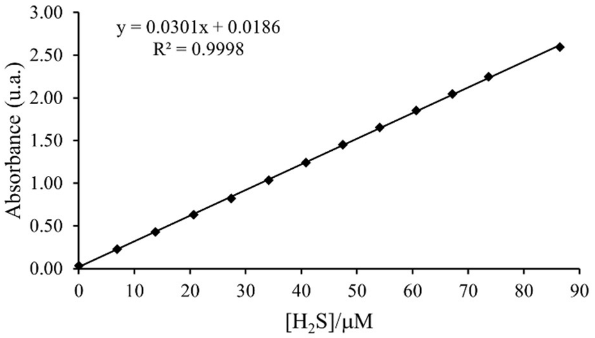

Figure S1. UV-Vis spectra of the DTNB (decreasing) and 5-thio-2-nitrobenzoate (increasing) (left) and calibration curve (right) obtained using a Na_2S solution.

S2. FTIR data

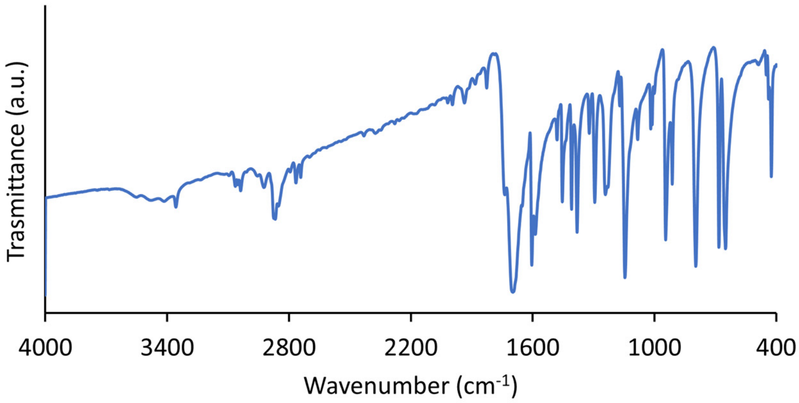

Figure S2. FTIR spectra of isophthalaldehyde.

S3. TG-DSC

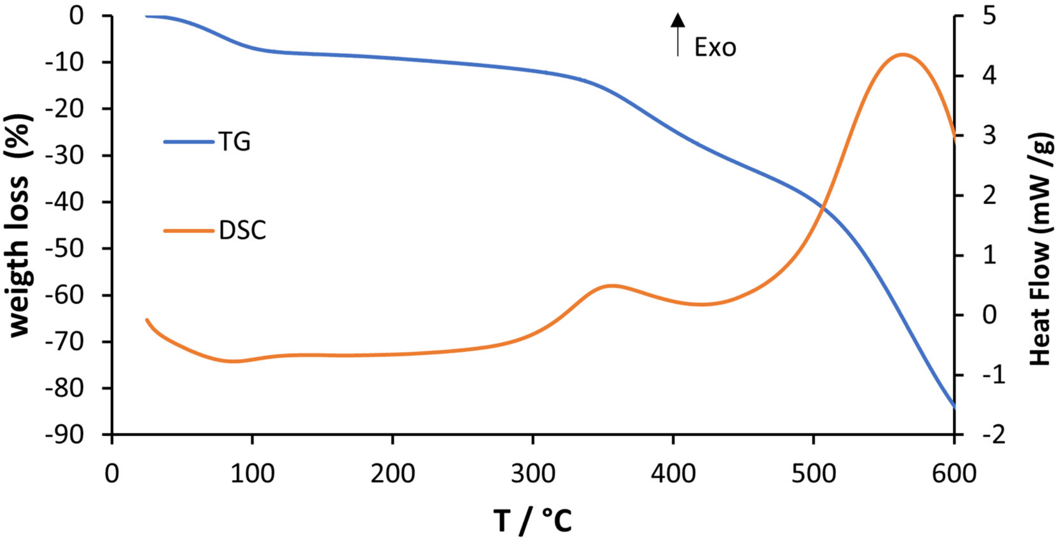

Figure S3. Thermogravimetry (blue line) and Differential Scanning Calorimetry (orange line) of the studied material.
